# Headache prevalence and demographic associations in the Delhi and National Capital Region of India: estimates from a cross-sectional nationwide population-based study

**DOI:** 10.1186/s10194-024-01814-2

**Published:** 2024-06-28

**Authors:** Debashish Chowdhury, Anand Krishnan, Ashish Duggal, Ritvik Amarchand, Andreas Husøy, Timothy J. Steiner

**Affiliations:** 1 GB Pant Institute of Postgraduate Medical Education and Research, New Delhi, India; 2 All India Institute of Medical Sciences, New Delhi, India; 3 Department of Neuromedicine and Movement Science, Norwegian University of Science and Technology (NTNU), Edvard Griegs gate, Trondheim, Norway; 4 Department of Neurology, University of Copenhagen, Copenhagen, Denmark; 5 Division of Brain Sciences, Imperial College London, London, UK

**Keywords:** Epidemiology, Prevalence, Population-based study, Headache, Migraine, Tension-type headache, Medication-overuse headache, India, South East Asia Region, Global Campaign against Headache

## Abstract

**Background:**

India is a large and populous country where reliable data on headache disorders are relatively scarce. This study in northern India (Delhi and National Capital Territory Region [NCR], including surrounding districts in the States of Haryana, Uttar Pradesh and Rajasthan) continues the series of population-based studies within the Global Campaign against Headache and follows an earlier study, using the same protocol and questionnaire, in the southern State of Karnataka.

**Methods:**

This cross-sectional study used the Global Campaign’s established methodology. Biologically unrelated Indian nationals aged 18–65 years were included through multistage random sampling in both urban and rural areas of NCR. Interviews at unannounced household visits followed the structured Headache-Attributed Restriction, Disability, Social Handicap and Impaired Participation (HARDSHIP) questionnaire in its original English version or in the validated Hindi version. Demographic enquiry was followed by a neutral headache screening question and diagnostic questions based on the International Classification of Headache Disorders edition 3 (ICHD-3), which focused on each respondent’s most bothersome headache. Questions about headache yesterday (HY) enabled estimation of 1-day prevalence. A diagnostic algorithm first identified participants reporting headache on ≥ 15 days/month (H15+), diagnosing probable medication-overuse headache (pMOH) in those also reporting acute medication use on ≥ 15 days/month, and “other H15+” in those not. To all others, the algorithm applied ICHD-3 criteria in the order definite migraine, definite tension-type headache (TTH), probable migraine, probable TTH. Definite and probable diagnoses were combined.

**Results:**

Adjusted for age, gender and habitation, 1-year prevalences were 26.3% for migraine, 34.1% for TTH, 3.0% for pMOH and 4.5% for other H15+. Female preponderance was seen in all headache types except TTH: migraine 35.7% vs. 15.1% (aOR = 3.3; *p* < 0.001); pMOH 4.3% vs. 0.7% (aOR = 5.1; *p* < 0.001); other H15 + 5.9% vs. 2.3% (aOR = 2.5; *p* = 0.08). One-day prevalence of (any) headache was 12.0%, based on reported HY. One-day prevalence predicted from 1-year prevalence and mean recalled headache frequency over 3 months was slightly lower (10.5%).

**Conclusions:**

The prevalences of migraine and TTH in Delhi and NCR substantially exceed global means. They closely match those in the Karnataka study: migraine 25.2%, TTH 35.1%. We argue that these estimates can reasonably be extrapolated to all India.

## Background

The background and rationale for undertaking this study have been discussed previously [1]. India is a large and extremely populous country where reliable data on headache disorders as a cause of population ill health are relatively scarce, and conflicting [2–4]. Since almost one in five people in the world reside in India [5], such data are of importance not only to health policy in India but also to understanding the global burden of headache.

This study continues the series of population-based studies supported by *Lifting The Burden* (LTB) within the Global Campaign against Headache [6], and is the second such study conducted in India. The first, in the southern State of Karnataka [2, 3, 7, 8], showed primary headaches to be both common and burdensome. However, India’s size, and cultural, ethnic and religious diversities, limit generalization from one State to the country. This study, in the northern Delhi and National Capital Territory Region (NCR), used the same protocol and questionnaire [9, 10] as all in the series. The principal purpose was to establish whether the prevalences of the headache disorders of public-health importance, and the burdens attributable to them, were similar in the north and in the south of the country. If so, the findings would be generalizable to the country, informing not only national health policy but also health policies in each State.

The study considered migraine, tension-type headache (TTH) and the group of disorders characterized by headache on ≥ 15 days/month (H15+), which include medication-overuse headache (MOH). This paper reports prevalence estimates and associations with demographic variables. Attributable burdens will be reported later.

## Methods

These have been described in detail previously [1]. We summarise them here.

### Ethics

The study was conducted in accordance with the Declaration of Helsinki [11]. The protocol and questionnaire were approved by the Institutional Ethics Committee of Maulana Azad Medical College and Associated Hospitals, New Delhi. All participants gave informed oral consent before enrolment.

All data were managed in accordance with data-protection legislation.

### Study design and sampling

This was a cross-sectional study of the adult population of Delhi and NCR, collecting data between December 2018 and June 2019, prior to the SARS-CoV-2 (Covid-19) pandemic. It used LTB’s established methodology [9, 10]. Biologically unrelated Indian nationals aged 18–65 years were eligible for inclusion. The study sampled both urban and rural areas of NCR, a region that encompasses the State of Delhi and surrounding districts in the States of Haryana, Uttar Pradesh and Rajasthan, with a total population of > 46 million and an urbanization level of 62.6% [12, 13].

A multistage random sampling approach was employed to achieve geographical and socioeconomic representativeness. Randomly selected households throughout the study areas were visited unannounced, and one adult member (aged 18–65) of each resident family selected for interview using the KISH method [14]. If the selected individual was not available at first visit, an appointment was made to return later. Those not able to complete the interview due to physical or mental health conditions, and immigrants, were considered ineligible and not counted as non-participants.

According to guidelines [9], we planned for a total sample of *N* ≥ 2,000 participants. We oversampled rural areas for sufficient numbers to analyse habitation as a potential associated variable.

Four interviewers fluent in Hindi and English conducted the survey. All had experience of community-based surveys and were given one week of specific training.

Interviews followed LTB’s structured Headache-Attributed Restriction, Disability, Social Handicap and Impaired Participation (HARDSHIP) questionnaire [10], in its original English version or in the validated Hindi version [1]. Demographic enquiry was followed by headache diagnostic questions based on ICHD-3 [15], which focused on each respondent’s most bothersome headache, diagnosing only this headache when more than one headache type was reported. Questions about headache yesterday (HY) enabled estimation of 1-day prevalence [10]. Additional questions about headache-attributed burden will be reported later.

Questionnaires were checked daily, and interviewers sent back to households next day when errors or missing entries were found. Other quality-control measures have been previously described [1].

Data were entered into Excel, with double data-entry of 50% of the questionnaires revealing few discrepancies (3.5%), all of which were resolved. A cross-check of 10% of the data against the original questionnaires raised no concerns, with an error rate of only 0.9%.

### Data analysis

#### Demographic variables

Gender was recorded as male or female. Age was recorded as a continuous variable, but later categorized as 18–25, 26–35, 36–45, 46–55 or 56–65 years. Habitation was recorded as either urban or rural. Marital status was recorded as single, married, separated or divorced/widowed, the last two groups combined for analyses. Education level was recorded as illiterate, primary school, middle school, high school, intermediate or post high school, graduate or postgraduate, or profession or honours. Annual household income was recorded in Indian rupees (INR) in six categories (< 150,000; 150,000-299,999; 300,000-499,999; 500,000-649,999; 650,000-999,999; ≥1,000,000) (on 31st May 2019, USD 1.00 = INR 69.60 [16]).

#### Headache diagnoses

All participants giving affirmative answers to the screening question (“did you have headache in the preceding year?”) were considered to have an active headache disorder [15]. Diagnoses were made by algorithm [10], which first identified participants reporting H15+. These were diagnosed as probable MOH (pMOH) if also reporting consumption of acute medication (assumed for the vast majority to be restricted to simple analgesics) on ≥ 15 days/month, or “other H15+” if not (with no further attempt at diagnosis [10]). To all others, the algorithm applied ICHD-based criteria [15] in the following order: those for definite migraine, definite TTH, probable migraine and, finally, those for probable TTH. Definite and probable diagnoses were combined in the association analyses. Remaining cases were unclassified.

#### Statistics

For demographic data, we summarized continuous variables as means with standard deviations (SDs) and categorical variables as percentages. We compared mean age, male-female ratio and urban-rural ratio of the sample with those of India’s population aged 18–65 years using t-test or chi-squared tests.

Prevalence estimates were reported as proportions (%) with 95% CIs. Age- and gender-adjusted 1-year prevalences of all headache and of each headache type were also calculated. Observed point prevalence of any headache (estimated through the question “did you have a headache yesterday?”) was compared with predicted point prevalence calculated from 1-year prevalence and reported mean headache frequency (days/month).

Associations between each headache type and demographic variables were investigated using bivariate and multivariate analyses. Odds ratios (ORs) and adjusted ORs (aORs) with 95% CIs were calculated.

We used RStudio 2023.6.2.561 for all analyses. We considered *p* < 0.05 to be significant. We did not adjust for multiple comparisons, for reasons explained in the Discussion.

## Results

### Description of sample

From 3,040 eligible households visited, a total of 2,066 individuals agreed to take part. The participating proportion (68.0% overall) was substantially higher in rural (98.3%) than in urban (52.9%) areas [1]. Females (64.3%) were overrepresented in comparison with the population gender distribution in India (48.3% female [17]; chi-squared = 209.8, *p* < 0.001). So, too, were urban dwellers in comparison with the nation as a whole (52.1% vs. 36% [17], chi-squared = 231.8, *p* < 0.001), but in comparison with the population of NCR (62.6% urban [12]) they were undersampled (chi-squared = 97.3, *p* < 0.001). As noted, this was deliberate (see Methods). Mean age in our sample was 38.8 years ± SD 13.2, close to but nonetheless significantly different from that of India’s population aged 18–65 years (mean = 37.7 years [17]; t(df = 2,065) = 3.4; *p* < 0.001).

### Lifetime prevalence

In total, 1,918 participants (92.8% [95% CI: 91.6–93.9]) reported ever having had (any) headache. The proportion was significantly higher among females (97.4% [96.4–98.2]) than males (84.5% [81.7–87.0]).

### One-year prevalence

One-year prevalence of (any) headache was 70.1%, again significantly higher among females (80.5% [78.3–82.6]) than males (51.3% [47.6–54.9]). Table 1 shows observed 1-year prevalence of each headache type, overall and by gender. Headache remained unclassified in only one participant. Overall, TTH was the most common headache type (34.1%), followed closely by migraine (28.3%). This difference was mainly due to a much lower prevalence of migraine among males (15.1%) than females (35.7%): migraine and TTH were equally prevalent among females (35.7% and 34.6% respectively) (Table 1). H15 + was reported by 7.6% of participants, and diagnosed as pMOH in 3.0%, other H15 + in 4.6%.

Since the sample did not match the population for age, gender or habitation, Table 1 shows prevalence estimates adjusted for all of these variables, with differences apparent only in all headache (67.9%) and migraine (26.3%).

**Table 1:** Observed and adjusted* one-year prevalence estimates by gender

Headache type	Observed			Adjusted* % [95% CI]
	**Overall** % [95% CI]	**Male** % [95% CI]	**Female** % [95% CI]	
All headache	70.1 [68.1–72.0]	51.3 [47.6–54.9]	80.5 [78.3–82.6]	67.9 [65.8–69.9]
Migraine	28.3 [26.4–30.3]	15.1 [12.6–17.9]	35.7 [33.1–38.2]	26.3 [24.4–28.3]
definite	18.7 [17.0-20.4]	8.4 [6.6–10.7]	24.4 [22.1–26.8]	
probable	9.6 [8.4–11.0]	6.6 [5.0-8.8]	11.3 [9.7–13.1]	
TTH	34.1 [32.1–36.2]	33.2 [29.9–36.8]	34.6 [32.1–37.2]	34.1 [32.1–36.2]
definite	29.5 [27.6–31.6]	28.4 [25.2–31.8]	30.2 [27.7–32.7]	
probable	4.6 [3.8–5.6]	4.9 [3.5–6.8]	4.4 [3.4–5.7]	
pMOH	3.0 [2.3–3.9]	0.7 [0.3–1.7]	4.3 [3.3–5.6]	3.0 [2.3–3.9]
Other H15+	4.6 [3.8–5.6]	2.3 [1.4–3.7]	5.9 [4.7–7.3]	4.5 [3.7–5.5]

*Adjusted for age, gender and habitation according to distributions in the national population; TTH: tension-type headache; pMOH: probable medication-overuse headache; H15+: headache on ≥ 15 days/month

### One-day prevalence

HY was reported by 17.1% of those reporting any headache in the preceding year (70.1%), yielding an overall 1-day prevalence of 12.0% [10.6–13.5]. The predicted 1-day prevalence, based on observed 1-year prevalence and reported mean headache frequency (4.5 days/month), was a little lower (10.5%). HY was more common among those with migraine (17.1%) than TTH (7.5%), and, as expected, much more common among those with pMOH (66.1%) or other H15+ (53.7%).

### Associations

Tables 2 and 3 show bivariate and multivariate analyses of associations between headache and the recorded demographic variables. All variables included in the bivariate analyses showed at least one significant association with one or more headache types, and were therefore also included in the multivariate models.

Female preponderance was confirmed in both analyses for all headache types except TTH: migraine 35.7% vs. 15.1% (aOR = 3.3; *p* < 0.001); pMOH 4.3% vs. 0.7% (aOR = 5.1; *p* < 0.001); other H15 + 5.9% vs. 2.3% (aOR = 2.5; *p* = 0.08).

Neither migraine nor other H15 + were associated with age in either analysis. TTH was least prevalent among those aged 46–55 years (OR = 0.6; *p* = 0.004), and pMOH more prevalent between ages 26 and 55 years in bivariate but not multivariate analyses.

Migraine (OR = 1.3; *p* = 0.003), pMOH (OR = 3.5; *p* < 0.001) and other H15+ (OR = 2.0; *p* = 0.001) were more common among rural dwellers in bivariate analysis, but only pMOH remained so in multivariate analysis (aOR = 2.4; *p* = 0.02). TTH was not associated with habitation.

There were other associations apparent only in bivariate analyses (Table 2). In particular, married participants had more migraine (OR = 1.4; *p* = 0.02) and pMOH (OR = 11.9; *p* = 0.01), but less TTH (OR = 0.7; *p* = 0.02) than single respondents; widowed, separated or divorced participants had more pMOH (OR = 9.8; *p* = 0.04) and other H15+ (OR = 3.0; *p* = 0.01); illiteracy was associated with migraine (OR = 2.3; *p* < 0.001), pMOH (OR = 11.2; *p* = 0.001) and other H15+ (OR = 7.6; *p* = 0.006); low household income was associated with migraine (OR = 1.8; *p* < 0.0001), pMOH (OR = 10.9; *p* = 0.02) and other H15+ (OR = 3.8; *p* = 0.01). In the multivariate analyses, TTH was variably associated with education level (Table 3).

**Table 2:** Bivariate analyses of associations between headache types and demographic variables

Demographic variable	Migraine	TTH	pMOH	Other H15+
	Odds ratios [95% CIs]			
**Gender**				
Male (*n* = 737)	reference	reference	reference	reference
Female (*n* = 1,329)	3.1 [2.5-4.0] **p < 0.001**	1.1 [0.9–1.3] *p* = 0.53	6.6 [2.9–18.9] **p < 0.001**	2.6 [1.6–4.6] **p < 0.001**
**Age** (years)				
18–25 (*n* = 401)	reference	reference	reference	reference
26–35 (*n* = 552)	1.2 [0.9–1.6] *p* = 0.15	1.0 [0.8–1.3] *p* = 0.92	3.2 [1.2–11.0] **p = 0.04**	1.2 [0.6–2.1] *p* = 0.63
36–45 (*n* = 479)	1.4 [1.0-1.8] *p* = 0.05	0.9 [0.6–1.1] *p* = 0.26	4.3 [1.6–15.0] **p = 0.008**	1.0 [0.5–1.8] *p* = 0.92
46–55 (*n* = 342)	1.2 [0.9–1.7] *p* = 0.24	0.6 [0.5–0.9] **p = 0.004**	3.6 [1.2–13.0] **p = 0.03**	0.9 [0.5–1.8] *p* = 0.82
56–65 (*n* = 292)	0.8 [0.5–1.1] *p* = 0.18	0.7 [0.5-1.0] *p* = 0.06	3.2 [1.0-11.7] *p* = 0.06	0.6 [0.3–1.4] *p* = 0.28
**Habitation**				
Urban (*n* = 1,076)	reference	reference	reference	reference
Rural (*n* = 990)	1.3 [1.1–1.6] **p = 0.003**	1.0 [0.8–1.2] *p* = 0.94	3.5 [2.0-6.6] *p* < 0.001	2.0 [1.3–3.1] *p* = 0.001
**Marital status**				
Single (*n* = 325)	reference	reference	reference	reference
Married (*n* = 1,605)	1.4 [1.1–1.9] **p = 0.02**	0.7 [0.6-1.0] **p = 0.02**	11.9 [2.6-211.3] **p = 0.01**	1.5 [0.8–3.1] *p* = 0.24
Widowed, separated or divorced (*n* = 136)	1.4 [0.9–2.1] *p* = 0.18	0.8 [0.5–1.2] *p* = 0.23	9.8 [1.4–193.0] **p = 0.04**	3.0 [1.3–7.4] *p* = 0.01
**Education level**				
Illiterate (*n* = 319)	2.3 [1.6–3.5] **p < 0.001**	1.4 [0.9-2.0] *p* = 0.12	11.2 [3.3–69.5] **p = 0.001**	7.6 [2.2–47.7] **p = 0.006**
Primary school (*n* = 67)	1.9 [1.0-3.4] **p = 0.04**	1.8 [1.0-3.2] **p = 0.04**	1.6 [0.1–17.3] *p* = 0.69	10.6 [2.4–73.4] **p = 0.004**
Middle school (*n* = 378)	1.7 [1.2–2.6] **p = 0.006**	1.5 [1.0-2.1] **p = 0.04**	3.8 [1.0-24.6] *p* = 0.08	6.0 [1.7–37.9] **p = 0.02**
High school (*n* = 327)	1.4 [0.9–2.1] *p* = 0.10	1.4 [1.0-2.1] *p* = 0.09	2.7 [0.7–18.0] = 0.21	6.6 [1.9–41.9] **p = 0.01**
Intermediate or post high school (*n* = 274)	1.2 [0.8–1.9] *p* = 0.33	1.9 [1.3–2.9] **p < 0.001**	0.8 [0.1–6.6] *p* = 0.81	3.2 [0.8–21.6] *p* = 0.14
Graduate or post graduate (*n* = 484)	1.3 [0.9-2.0] *p* = 0.15	1.6 [1.2–2.4] **p = 0.006**	1.3 [0.3–9.3] *p* = 0.72	4.4 [1.3–27.7] *p* = 0.05
Profession or honours (*n* = 217)	reference	reference	reference	reference
**Household income** (INR)^1^				
< 150,000 (*n* = 632)	1.8 [1.3–2.7] **p < 0.001**	1.3 [0.9–1.9] *p* = 0.10	10.9 [2.3-195.1] **p = 0.02**	3.8 [1.5–12.7] **p = 0.01**
150,000-299,999 (*n* = 562)	1.3 [0.9-2.0] *p* = 0.13	1.6 [1.2–2.3] **p = 0.006**	6.4 [1.3-115.6] *p* = 0.05	2.6 [1.0–9.0] *p* = 0.07
300,000-499,999 (*n* = 350)	1.3 [0.9–1.9] *p* = 0.25	1.4 [1.0-2.1] *p* = 0.06	4.7 [0.8–86.7] *p* = 0.15	1.7 [0.6–6.3] *p* = 0.34
500,000-659,999 (*n* = 139)	0.7 [0.4–1.2] *p* = 0.21	2.0 [1.2–3.1] **p = 0.004**	3.0 [0.3–64.8] *p* = 0.37	1.1 [0.2–5.1] *p* = 0.89
650,000-999,999 (*n* = 164)	0.9 [0.5–1.5] *p* = 0.63	1.2 [0.8–1.9] *p* = 0.42	2.5 [0.2–54.7] *p* = 0.45	1.3 [0.3–5.4] *p* = 0.74
> 1,000,000 (*n* = 206)	reference	reference	reference	reference

TTH: tension-type headache; pMOH: probable medication-overuse headache; H15+: headache on ≥ 15 days/month; ^1^3 participants missing; significant p-values are emboldened

**Table 3:** Multivariate analyses of associations between headache types and demographic variables

Demographic variable	Migraine	TTH	pMOH	Other H15+
	Adjusted odds ratios [95% CIs]			
**Gender**				
Male	reference	reference	reference	reference
Female	3.3 [2.6–4.2] **p < 0.001**	1.1 [0.9–1.3] *p* = 0.41	5.1 [2.1–15.3] **p < 0.001**	2.5 [1.5–4.6] **p = 0.001**
**Age** (years)				
18–25	reference	reference	reference	reference
26–35	1.1 [0.8–1.6] *p* = 0.48	1.2 [0.8–1.6] *p* = 0.38	1.9 [0.6–7.3] *p* = 0.30	0.9 [0.4–1.9] *p* = 0.76
36–45	1.4 [1.0-2.1] *p* = 0.09	1.0 [0.7–1.5] *p* = 0.85	2.4 [0.8–9.5] *p* = 0.16	0.8 [0.4–1.8] *p* = 0.55
46–55	1.4 [0.9–2.2] *p* = 0.10	0.8 [0.5–1.2] *p* = 0.22	2.1 [0.6–9.2] *p* = 0.26	0.8 [0.3–1.9] *p* = 0.59
56–65	0.9 [0.5–1.4] *p* = 0.52	0.9 [0.6–1.4] *p* = 0.71	1.9 [0.5–8.7] *p* = 0.34	0.5 [0.2–1.3] *p* = 0.17
**Habitation**				
Urban	reference	reference	reference	reference
Rural	1.2 [1.0-1.6] *p* = 0.10	0.9 [0.7–1.2] *p* = 0.49	2.4 [1.2–5.2] **p = 0.02**	1.6 [0.9–2.7] *p* = 0.09
**Marital status**				
Single	reference	reference	reference	reference
Married	0.9 [0.6–1.3] *p* = 0.61	0.8 [0.6–1.1] *p* = 0.21	3.1 [0.5–61.1] *p* = 0.30	1.4 [0.6–3.5] *p* = 0.41
Widowed, separated or divorced	0.6 [0.4–1.1] *p* = 0.13	0.9 [0.5–1.6] *p* = 0.76	1.3 [0.1–29.5] *p* = 0.82	2.6 [0.8–7.9] *p* = 0.09
**Education level**				
Illiterate	1.2 [0.7–1.9] *p* = 0.59	1.4 [0.9–2.2] *p* = 0.20	2.4 [0.5–18.6] *p* = 0.35	3.0 [0.7–21.2] *p* = 0.19
Primary school	1.3 [0.6–2.5] *p* = 0.51	1.8 [0.9–3.3] *p* = 0.08	0.5 [0.0-6.4] *p* = 0.60	5.4 [1.0-41.2] *p* = 0.06
Middle school	1.1 [0.7–1.8] *p* = 0.75	1.4 [0.9–2.2] *p* = 0.14	1.2 [0.2–9.1] *p* = 0.86	2.9 [0.7–19.6] *p* = 0.20
High school	1.1 [0.7–1.8] *p* = 0.67	1.2 [0.8–1.9] *p* = 0.32	1.3 [0.3–9.9] *p* = 0.79	4.2 [1.1–28.7] *p* = 0.07
Intermediate or post high school	1.1 [0.7–1.8] *p* = 0.74	1.6 [1.1–2.5] **p = 0.02**	0.5 [0.1–4.7] *p* = 0.51	2.3 [0.5–16.3] *p* = 0.32
Graduate or post graduate	1.3 [0.9–1.8] *p* = 0.20	1.5 [1.0-2.2] **p = 0.03**	1.1 [0.2-8.0] *P* = 0.93	3.8 [1.1–24.9] *p* = 0.08
Profession or honours	reference	reference	reference	reference
**Household income** (INR)				
< 150,000	1.5 [0.9–2.5] *p* = 0.10	1.2 [0.8–1.9] *p* = 0.46	3.6 [0.5–74.3] *p* = 0.28	1.5 [0.5-6.0] *p* = 0.52
150,000-299,999	1.1 [0.7–1.8] *p* = 0.65	1.4 [0.9–2.2] *p* = 0.10	3.0 [0.4–62.1] *p* = 0.34	1.2 [0.4–4.8] *p* = 0.73
300,000-499,999	1.1 [0.7–1.8] *p* = 0.66	1.3 [0.8–1.9] *p* = 0.27	2.4 [0.3–48.6] *p* = 0.46	0.9 [0.3–3.7] *p* = 0.91
500,000-659,999	0.6 [0.4–1.2] *p* = 0.15	1.8 [1.1–2.9] **p = 0.02**	2.2 [0.2–49.7] *p* = 0.55	0.8 [0.1–3.6] *p* = 0.72
650,000-999,999	0.7 [0.4–1.3] *p* = 0.28	1.1 [0.7–1.8] *p* = 0.65	2.2 [0.2–48.0] *p* = 0.53	0.9 [0.2–3.9] *p* = 0.88
> 1,000,000	reference	reference	reference	reference

TTH: tension-type headache; pMOH: probable medication-overuse headache; H15+: headache on ≥ 15 days/month; significant p-values are emboldened

## Discussion

This second study in India using LTB’s standardized methodology [9, 10] found primary headache disorders and pMOH to be common in the north of the country. Adjusted for age, gender and habitation, 1-year prevalences were 26.3% for migraine, 34.1% for TTH, 3.0% for pMOH and 4.5% for other H15+. One-day prevalence of (any) headache was 12.0%, based on reported HY (presumed to be free from recall error). One-day prevalence predicted from 1-year prevalence and mean recalled headache frequency over 3 months was slightly lower (10.5%).

These findings are well in excess of best estimates of global means: migraine 14–15% [18–20], TTH 26–27% [19, 20], pMOH 1–2% [21–24].

Of especial interest are comparisons with our previous study in the southern Indian State of Karnataka, using very much the same methodology [2, 3, 7, 8]. The Karnataka study based its diagnoses on ICHD-II [24], current at the time, whereas in the present study they were based on ICHD-3 [15]. With respect to the headache disorders of interest, no material differences exist between these two versions of ICHD. In the Karnataka study, the 1-year age-standardized prevalence of migraine was 25.2%, of TTH 35.1% [3], almost identical to our estimates for Delhi and NCR in the north.

At the time of the Karnataka study, the estimate of 25.2% for migraine was considered remarkable, because it was well above the estimated global prevalence of 14.7% [25], but believed nevertheless to be reliable [3]. The present study corroborates that finding, but, meanwhile, other LTB studies in countries around the world, using very similar methodology [9, 10], have put it within the global range [3, 26–35]. All of these studies included both definite and probable migraine.

There were, however, significant differences in H15 + between the two regions, here reported by 7.5% (pMOH 3.0% [95% CI: 2.3–3.9]; other H15 + 4.5% [3.7–5.5]), in Karnataka by only 3.0% (pMOH 1.2% [0.8–1.7]; other H15 + 1.7% [1.3–2.3] [3]). Other H15 + may include chronic migraine, chronic TTH, other relatively rare primary headache disorders and a range of secondary headaches, notably post-traumatic headache and headache attributed to communicable disorders (including malaria). Cross-sectional studies with a single encounter with each participant cannot reliably make these diagnoses [9, 10]; they therefore fall outside the scope of these studies, and there would be no purpose in speculating on these differences. pMOH on the other hand is within their scope. Although the diagnosis relies on the association of H15 + with reported acute medication on ≥ 15 days/month, without proof of causation (hence is only probable), the difference between north and south is large enough to suggest it is real. The genesis of MOH is complex, behaviourally conditioned by culture, education and socioeconomic circumstances [22] and strongly influenced by availability and quality of health care and ease of access to over-the-counter (OTC) medications, all likely to vary throughout India. Time may also be a factor, since the study in Karnataka was completed 12 years ago [9], but any influence of this would be small.

Associations were also similar between the two studies. As expected, headache in the present study was more common among females than males. In fact, headache seemed to have been a near universal experience for adult females in this region of India, with a lifetime prevalence (prior to the SARS-CoV-2 pandemic) of 97.4% (84.5% in males). Migraine, pMOH and other H15 + were all significantly more common in females than males. TTH, on the other hand, was equally prevalent in both genders. This gender-pattern was exactly as observed in Karnataka [3].

pMOH in the present study was more common in rural areas (aOR = 2.4). This association was similarly evident in Karnataka (aOR = 2.1 [3]), despite the difference in overall prevalence, and appears, therefore, to be robust. A plausible explanation invoked in the Karnataka study, and probably equally applicable here and throughout India [36], was less easy access in rural areas to health care but no great difficulty in obtaining OTC analgesics – overuse of these being the most common cause of MOH [22]. The lower literacy rate in rural areas [37], impeding self-education, was a likely contributing factor.

The associations in the bivariate analyses between most headache types and marital status were almost certainly confounded by age, and did not survive in the multivariate analyses.

Multivariate analyses showed little else of interest. Age, marital status and socioeconomic status (assessed from education level and household income) had little evident influence here on headache prevalence. In Karnataka, there was a limited effect of age on migraine prevalence, maximal (at about 28.5%) between ages 26 and 45 years, and no effect of household income.

As noted in Methods, we did not make adjustments in the association analyses for multiple comparisons. The number was not excessive (19 different comparisons in the bivariate analysis). Additionally, many of the tests were not independent of each other. Rather, we followed recommendations not to correct [38, 39], but instead report all individual p-values and confidence intervals, advocating cautious interpretation of p-values barely crossing the threshold of significance, especially in the bivariate analyses.

### Generalization to all India

These two sets of findings from Delhi/NCR in the north of India and Karnataka in the south, highly concordant with regard to migraine and TTH, are of major importance, because they support generalization to all India despite this country’s diversities of culture and climate. They provide a reasonably reliable indication that, in a population of 870 million aged 18–65 years (2020 estimate [40]), some 217 million people have migraine and a further 301 million have TTH.

However, a study in Kolkata, in the east of India with a reported population of 4.58 million, discordantly reported a 1-year prevalence of migraine of 14.1% (95% CI: 12.7–15.6) [4]. Conducted in 2011 but published in 2017, this study adopted a methodology similar in many respects to ours, with a “stratified random sampling strategy”, visits to 2,421 households and a neutral screening question (“Have you experienced headache in the last one year?”) [4]. Diagnosis of migraine was based on ICHD-II (as it was in the Karnataka study). However, multiple reasons arise for questioning its prevalence estimate, and for discounting the Kolkata study as unreliable. First, the participating proportion was not reported. While 436 “headache-positive subjects” were identified in the 2,421 households, only 374 were evaluated (62 “headache-positive subjects” were excluded). Second, while the study selected participants aged 20–50 years only, no account was given of the age and gender distributions in the sample [4], and there was no adjustment for these important variables [9]. Third, the manuscript makes no reference to probable migraine, and almost certainly the 14.1% was for definite migraine only. Fourth, the Kolkata study found an overall prevalence of “primary headache” of 14.9%, meaning, unfeasibly, that all cases of probable migraine and of TTH accounted for only 0.8%.

### Strengths and limitations

This study was carried out using established methodology [9, 10] in a sufficiently large sample drawn from the general population of Delhi/NCR. Pre-pilot and pilot studies, reported previously [1], had been performed to ensure study feasibility, with necessary adjustments made. Quality-control measures, also reported previously [1], were in place, and the diagnostic question set had been validated [1]. These were strengths.

Some limitations were also present, many inherent and to a large degree unavoidable in studies of this type. One in particular was that our aim for a sample representative of the region with regard to habitation and socioeconomic status (as well as age and gender) was hindered by a low participating proportion (68.0% overall), worse in urban areas (52.9%) [1]. Likely reasons, including scepticism regarding data privacy and unwillingness to participate in studies offering no personal benefit, both predictably more pronounced in high-income urban areas, have been discussed previously [1]. Whatever the explanation, this undermined our association analysis with regard particularly to pMOH and habitation, although it found much the same as was reported in Karnataka.

Enquiries regarding the preceding 3 months have built-in potential for recall error, which we countered by additional enquiry into headache yesterday. There was reasonable concordance between observed 1-day prevalence, based on reported HY, and predicted 1-day prevalence, based on 1-year prevalence and headache frequency recalled over 3 months.

Only one diagnosis was allowed in each participant, with focus on the most bothersome headache. In those with both migraine and TTH, the latter was more likely to be overlooked.

## Conclusion

The prevalences of the headache disorders of public-health importance in Delhi and National Capital Region of India substantially exceed global means. Adjusted for age, gender and habitation, 1-year prevalences were 26.3% for migraine, 34.1% for TTH, 3.0% for pMOH and 4.5% for other H15+. The findings for migraine and TTH closely match those of a similar study in Karnataka State in the south of India, and we argue that they can reasonably be extrapolated to all India.

While these estimates strongly suggest that headache disorders in India are of major concern, health policy also needs estimates of attributed burdens – of lost health, impaired participation and financial losses. These will be provided in a later manuscript.

## Data Availability

The original data are held on file at the All India Institute of Medical Sciences, New Delhi, India, and the analytical subset at Norwegian University of Science and Technology, Trondheim, Norway. Once analysis and publications are completed, they will be freely available for non-commercial purposes to any person requesting access in accordance with the general policy of the Global Campaign against Headache.

## Abbreviations

aOR: Adjusted odds ratio
CI: Confidence interval
H15+: Headache on ≥ 15 days/month
HARDSHIP: Headache-Attributed Restriction, Disability, Social Handicap and Impaired Participation (questionnaire)
HY: Headache yesterday
ICHD: International Classification of Headache Disorders
INR: Indian Rupee
LTB: Lifting The Burden
MOH: Medication-overuse headache
NCR: National Capital Region of Delhi
OR: Odds ratio
OTC: Over-the-counter
pMOH: Probable MOH
SD: Standard deviation
TTH: Tension-type headache
USD: United States dollar
